# Effect of an online Workplace Vocal Health and Low Stress Levels Promotion Program implemented in a Colombian university during COVID-19 pandemic

**DOI:** 10.1590/2317-1782/20232022052

**Published:** 2023-09-01

**Authors:** Ángela Patricia Atará-Piraquive, Leidy Lorena Forero-Suárez, Jenny Fernanda Cárdenas-Martinez, Lady Catherine Cantor-Cutiva

**Affiliations:** 1 Departamento de Salud de Colectivos, Facultad de Enfermería, Universidad Nacional de Colombia - Bogotá, Colombia.; 2 Programa de Fonoaudiología, Facultad de Medicina, Universidad Nacional de Colombia - Bogotá, Colombia.; 3 Communicative Sciences and Disorders Department, Michigan State University - East Lansing (MI), United States of America.

**Keywords:** Workplace, COVID-19, Faculty, Occupational Health, Health Promotion, Voice Quality, Local de Trabalho, Covid-19, Docentes, Saúde do Trabalhador, Promoção da Saúde, Qualidade da Voz

## Abstract

**Purpose:**

To determine the effect of an online Workplace Vocal Health and Low Stress Levels (WVHLS) Promotion Program implemented in a Colombian university during COVID-19 pandemic.

**Methods:**

This research was a quasi-experimental study. Twenty-nine professors participated in this study within two groups: (1) intervention group (n=17) or (2) non-intervention group (n=12). Participants in the intervention group took part in four virtual sessions about how to improve vocal health and strategies to reduce stress levels during their homeworking and online classes. Teachers filled in a questionnaire including questions about working conditions, work-related stress, and the voice functioning (including the Vocal Fatigue Index-VFI). They also recorded a voice sample of a sustained vowel on two separate occasions (before and after the follow-up).

**Results:**

At the end of the follow-up, there was a tendency to reduce Factor 1 of VFI in the intervention group. Although, all participants had a longer MPT at the end of the study compared with the baseline measures, males in the intervention group had longer MPT compared with other participants.

**Conclusion:**

Our results suggest a positive effect of a WVHLS promotion program on reducing vocal fatigue perception measured by means of the Vocal Fatigue Index and improving coordination and control of breathing speech measured MPT. These changes at the end of the follow-up may indicate that holistic programs that include voice care recommendations, breathing exercises, vocal warm-up, cold-down and laryngeal relaxation vocal exercises, and stress management may be beneficial for reducing work-related stress and voice symptoms among professors.

## RESUMO

**Objetivo:**

Determinar o efeito de um programa online de saúde vocal no local de trabalho e de redução dos níveis de estresse (SVLTRNE, ou por sua sigla em inglês, WVHLS - Workplace Vocal Health and Low Stress Levels), implementado em uma universidade colombiana durante a pandemia da COVID-19.

**Método:**

O desenho do estudo foi quase experimental, com a participação de vinte e nove professores, separados em dois grupos: grupo intervenção (n=17) e grupo controle (n=12). Os participantes do grupo de intervenção participaram de quatro sessões virtuais que abordaram como melhorar a saúde vocal e as estratégias para reduzir os níveis de estresse durante o trabalho. Os professores preencheram um questionário que incluía questões sobre condições de trabalho, estresse relacionado ao trabalho e índice de fadiga vocal (IFV). Também foi gravada uma amostra de voz com uma vogal sustentada, em duas ocasiões diferentes (antes e depois da intervenção).

**Resultados:**

No final desse estudo, foi observada uma tendência à redução do Fator 1 do IFV no grupo intervenção. Embora todos os participantes tivessem um TMF mais longo no final do estudo em comparação com as medidas basais, os homens do grupo de intervenção tiveram TMF mais longo em comparação com outros participantes.

**Conclusão:**

Os resultados deste estudo sugerem um efeito positivo do programa de promoção WVHLS na redução da percepção de fadiga vocal e na melhora da coordenação e controle da coordenação pneumofônica, medidos com o IFV e TMF. Essas mudanças, observadas ao final do estudo, podem indicar que programas holísticos que incluem recomendações de cuidados com a voz, exercícios respiratórios, exercícios vocais de aquecimento e resfriamento, exercícios vocais de relaxamento laríngeo e controle do estresse podem ser benéficos para reduzir o estresse relacionado ao trabalho e sintomas vocais em professores.

## INTRODUCTION

Teachers have been identified as an occupational voice group with a high risk of suffering from voice disorders. A systematic review of literature reported that the prevalence of current voice disorders among school-teachers ranged from 9% to 37%^(1)^, whereas college professors had prevalence between 35% and 45%^(2)^. Likewise, high-stress levels are common among teachers^(3,4)^. Gomes et al.^(5)^ reported that mental and emotional illnesses are highly prevalent in teachers due to highly stressful work environments and high workloads^(5)^. High-stress levels among teachers affect their health and well-being^(6,7)^, as well as teachers' motivation, job satisfaction and work ability^(7)^. In addition, professors with voice disorders reported higher stress levels compared to their vocally healthy colleagues^(8)^. Stress in teachers has been also associated with the duration and intensity of voice use (number of teaching years, full-time working, number of pupils)^(9)^.

College professors have an extra load due to the mixing between teaching, research, and service with administrative tasks^(10)^, which can increase their vocal demands and stress levels. Moreover, during COVID-19, college professors faced significant stressors and increased the likelihood of reporting hoarseness due to changes in their working conditions that included teaching online classes (often without previous knowledge of virtual environments)^(11)^. The vocal demand of online teaching implies speaking for long periods with restricted communicative interaction with their students^(12)^, the presence of external noise sources^(13)^, incorrect posture when teaching classes, inappropriate habits, lack of hydration^(14)^, and poor ergonomics causing neck pain^(15)^. A recent study reported that teachers who raised their vocal loudness during online teaching experienced significantly higher-stress levels^(16)^. In addition, productivity reduction costs in teachers associated with high stress levels were around US$8,210 during pandemic COVID-19^(17)^.

Recently, it has been highlighted the importance of implementing workplace vocal and mental health programs in educational settings to reduce the occurrence of voice disorders and stress levels among teachers^(13,17)^. However, there is a lack of studies that report workplace programs to improve teachers’ quality of life and voice functioning^(18)^. Workplace settings are optimal for implementing health promotion programs because they can offer the appropriate structure, environment and social setting^(19)^. Moreover, workplace health promotion interventions may target psychosocial and physical factors, which will allow them to reduce health risks and improve productivity^(20)^. A previous study reported higher voice-related quality of life after a workplace vocal health promotion program^(10)^, which highlight the effect of implementing activities in the workplace that promote vocal health and reduce stress levels among college professors. Furthermore, 100% online interventions have been found to be effective for increasing knowledge about vocal health and vocal techniques (warm-up and cool-down), reducing the presence of voice symptoms and improving the voice quality of teachers after 4 workshops^(14)^.

Therefore, this paper aims to analyze the effect of an online Workplace Vocal Health and Low Stress Levels (WVHLS) Promotion Program implemented among college professors.

## METHODS

### Study design and participants

This quasi-experimental study was conducted at a Colombian university with data collection performed from September 2021 to December 2021. Our hypothesis was that the WVHLS program would decrease stress levels measured by means of self-administrated questionnaires, as well as improve voice quality measured with the V-RQOL, the VFI, and voice acoustic parameters. The invitation to participate was disclosed via email through the Dean’s offices. The week before starting the first session, the second author and the participant met via Zoom to fill in the informed consent form, the baseline questionnaire and recorded the baseline voice sample. This study was approved by the Medical Ethics Committee of the Universidad Nacional de Colombia (approval code 006−21). Participants were assigned to each group based on their entry time into the study (intervention group = first 17 participants, and non-intervention group = last 12 participants).

#### Inclusion and exclusion criteria

Four inclusion criteria were defined: First, participants should be professors from Universidad Nacional de Colombia. Second, they should have electronic devices (cellphone, computer, tablet) and an internet connection to access the questionnaires, record the voice samples and attend the online sessions. Third, participants should not have received any voice training or stress therapy. Participants were excluded if they were participating in similar studies. Fourth, persons with chronic voice disorders were excluded from this study. This criterion was defined because people with these conditions may have received voice therapy with a further effect than vocal health promotion programs. These programs are part of primary prevention actions, which aim to prevent disease or injury before it ever occurs; whereas voice therapy is a tertiary prevention action that aims to soften the impact of an ongoing illness or injury that has lasting effects. Since this study was implemented during COVID-19 pandemic and our participants were still home working due to quarantines, it was not possible to perform clinical examinations. Therefore, chronic voice disorders were defined by means of the self-report filled in by each professor.

### Data collection procedures

The duration of this study was five weeks. Before starting the program, the second author delivered the instruments (microphone, adapter, and earphones) at the participant’s home. Then, the researchers and the participant met via Zoom to fill-in the informed consent form, the baseline questionnaire “*Questionnaire of work-related voice and stress health*” and recorded the baseline voice sample.

After the baseline assessment, professors from the intervention group participated in the four-week WVHLS promotion program. Each virtual session was performed on a weekly basis and included instructions for daily practice exercises. Participants in the non-intervention group did not participate in the intervention program until the data collection process was finished. Once the data collection was finished, these professors had full access to the virtual sessions and all the material of the WVHLS promotion program.

#### Questionnaire of work-related voice and stress health

For this study, we designed a questionnaire, with six sections, based on previous publications^(12,20)^, and including questions about factors previously identified as associated with voice production and stress levels among teachers. The first part of the questionnaire contained questions about sociodemographic information (age, gender, and marital status). No cut-offs were defined because the purpose was to assess the distribution of independent factors in relation to voice production and stress levels. The second part included questions about psychosocial factors. We included questions related to quality of sleep^(21)^, and self-perceived stress levels^(22)^. The stress question was: “Stress means a situation in which a person feels tense, restless, nervous, or anxious, or is unable to sleep at night because his/her mind is troubled all the time. ¿Do you feel this kind of stress currently?” (Spanish version: El estrés es definido como una situación en la cual una persona se siente tensa, inquieta, nerviosa, ansiosa o es incapaz de dormir en la noche porque su mente está preocupada todo el tiempo. ¿Siente este tipo de estrés actualmente?). A Likert scale was used to answer this item where *1* means *not at all* and *5* mean *very much* (as in the original version)^(22)^. The third part of the questionnaire included questions about voice functioning. The fourth section included the Vocal Fatigue Index (VFI) validated in Spanish by Cantor-Cutiva et al.^(23)^, which contains 19 statements, divided into three factors: tiredness of voice and voice avoidance, physical discomfort associated with voicing, and improvement of voice symptoms with rest. The VFI is an instrument that allows to identify speakers with vocal fatigue. For Factor 1 and 2, higher scores reflect higher vocal fatigue, whereas for Factor 3 lower scores are related with higher vocal fatigue. Factor 1 is rated from 0 to 44 with a cut-off value of 9, factor 2 is rated from 0 to 20 and the cut-off value is 1.5, and factor 3 is rated from 0 to 12 with a cut-off value of 9. The fifth part included the Voice-Related Quality of Life (V-RQOL), which includes questions related to physical and socio-emotional functioning, rated on a Likert scale from *1=None, I have no problem* to *5=Always*^(24)^. The V-RQOL assesses voice functioning in relation to individual and environmental factors. Raw scores on the V-RQOL range from 10 to 50 and are converted using an algorithm to a scale from 0 to 100, where 0 indicates the worst quality of life and 100 indicates no impact on quality of life. The sixth part of the questionnaire included questions about costs associated with voice problems and stress. Section two (psychosocial factors) and four (VFI) were applied before and after the program to determine if stress levels and voice symptoms changed after the intervention.

#### Voice recordings

Voice samples were recorded online during a video call via *Zoom*. Recordings were made using a TrustMico 20378 microphone and following the American Speech, Language and Hearing Association (ASHA) recommendations^(25)^. Before starting this procedure, participants were asked to download *Praat* Software^(26)^ in their laptops. Then, at the beginning of the session, the researcher and the participant checked that the microphone and headphones were correctly plugged into the computer. If everything was working properly, participants were requested to activate the remote control on *Zoom* to allow the researcher to record the voice samples using Praat. For voice recordings, participants were asked to produce three times a sustained vowel /a/ for as long as possible at a conversational pitch and loudness. Voice acoustic analysis was performed by selecting five seconds of the third sustained vowel /a/ and registering the longest duration to extract maximum phonation time (MPT). For voice analysis, we extracted Jitter Local, Shimmer Local and Harmonics to Noise Ratio (HNR). Reference values were based on Delgado et al.^(27)^ who analyzed the acoustic parameters of Spanish speakers. Reference values were as follows^(1)^: for jitter, between 0.18 and 0.72^(2)^, for Shimmer between 0.47 and 3.38, and^(3)^ for HNR between 18.71 and 29.87^(27)^.

### Workplace Vocal Health and Low Stress Levels Promotion Program

This program included four virtual weekly sessions. Each session included four modules^(1)^: Improvement of working conditions^(2)^, stress management^(3)^, voice care recommendations and voice training, and^(4)^ promotion of healthy lifestyle habits for teachers (Table 1). The week before starting the first session, the researcher and the participant met via *Zoom* to fill-in the informed consent form, the baseline questionnaire and recorded the baseline voice sample. The following weeks, each participant in the intervention group received and email with the link for the individual sessions of the program, which were developed via Zoom. The duration of these sessions was approximately 45 minutes. During the sessions, both researcher and participant had their cameras active (ON) to facilitate monitoring and feedback. In addition, participants were instructed to maintain a sitting posture during the sessions, and drink water as needed. After finishing the last session, the researcher and the participant met via *Zoom* to fill-in the shorter version of the questionnaire and recorded the voice sample.

**Table 1:** Description of the modules: Workplace Vocal Health and Low Stress Levels Promotion Program

MODULE – INFORM YOURSELF
A forum was built in google classroom for the participants to answer three questions about stress management, vocal health care, and the institution's tools and infrastructure. These questions were designed with the purpose of sharing knowledge and opinions among the participating teachers regarding this aspect.
MODULE II – STRESS MANAGEMENT
Strategies or activities on how to organize time were shared with the subjects, a meditation was included in a 10-minute recording, exercises to take work breaks during the workday to reduce stress levels.
MODULE III – VOCAL HEALTH CARE
Videos were made explaining how to perform vocal warm-up and cold-down exercises, and laryngeal relaxation vocal exercises, breathing exercises for teachers to apply in their workplaces.
MODULE IV – PROMOTION OF HEALTHY HABITS
Topics on the importance of taking healthy breaks and having an organized workplace were addressed. In addition, videos were shared with visual work breaks, blinking exercises, hand care exercises, and short exercises for shoulders, neck, and head to perform during the workday. Infographics were also used so that teachers had direct access to the information.

**Figure 1:**
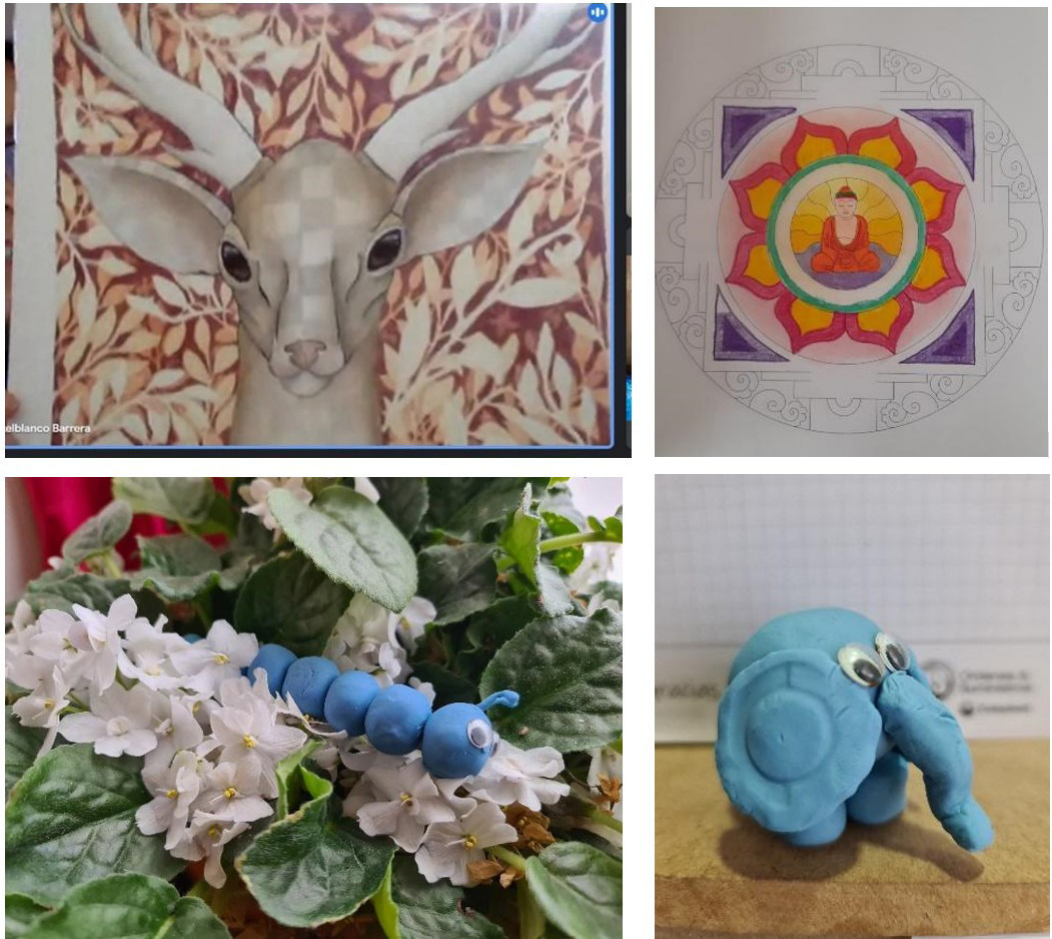
Artistic pieces created by the participants of the intervention group

Figure 1 shows the results of one of the activities in the third session - module II for stress management. This activity consisted of creating an artistic piece to externalize the feelings or emotions that the participants had. For this, they were requested to create a drawing, make a figure in clay or paint.

### Statistical analysis

All statistical analyses were performed using SPSS 25 (IBM Corporation). Dependent variables were the VFI scores, stress level, hours per day of voice use, Shimmer local, Jitter Local, HNR and MPT. The independent variable was participation in the WVHLS Promotion Program (yes/no). Normal distribution of the data was assessed using the Shapiro-Wilk test. Descriptive statistics were used to characterize the study population. We used the Wilcoxon signed-rank test to assess differences in the dependent variables before and after the implementation of the program. Also, we determined the causal association between and effect the dependent variables and the independent variables through the Generalized Estimating Equations (GEEs). All the significance levels were established at p<0.05.

## RESULTS

### Participant characteristics

In total, 29 participants were recruited for this study. Sociodemographic characteristics are presented in Table 2. Participants were 13 females and 16 males. Seventeen subjects were assigned to the intervention group and twelve participants to the non-intervention group. On average, professors in the intervention group were 48 years old (SD= 10.4), whereas participants in the non-intervention group were 40 years old (SD=10.8). Participants in both groups had similar hours of vocal use and teaching days per week (Table 3).

**Table 2:** Characteristics of 29 participating college professors

ID	Group	Gender	Age	Academic category	Faculty
L01	Intervention	F	34	Instructor	Library
L02	Intervention	F	55	Instructor	Library
L03	Intervention	F	54	Associate professor	Nursing
L04	Intervention	F	37	Adjunct professor	Human Sciences
L05	Intervention	F	46	Associate professor	Human Sciences
L06	Intervention	F	36	Assistant professor	Human Sciences
L07	Intervention	F	35	Associate professor	Human Sciences
L08	Intervention	F	46	Adjunct professor	Human Sciences
L09	Intervention	M	48	Associate professor	Dentistry
L10	Intervention	M	42	Assistant professor	Engineering
L11	Intervention	F	49	Associate professor	Human Sciences
L12	Intervention	F	40	Assistant professor	Engineering
L13	Intervention	F	57	Associate professor	Medicine
L14	Intervention	F	49	Adjunct professor	Economic Sciences
L15	Intervention	M	75	Full professor	Medicine
L16	Intervention	F	49	Adjunct professor	Economic Sciences
L17	Intervention	M	55	Associate professor	Sciences
L18	Control	M	44	Associate professor	Economic Sciences
L19	Control	M	41	Adjunct professor	Economic Sciences
L20	Control	F	62	Instructor	Library
L21	Control	M	29	Adjunct professor	Human Sciences
L22	Control	F	41	Adjunct professor	Economic Sciences
L23	Control	F	53	Associate professor	Sciences
L24	Control	M	30	Adjunct professor	Human Sciences
L25	Control	M	30	Adjunct professor	Economic Sciences
L26	Control	F	33	Adjunct professor	Economic Sciences
L27	Control	F	28	Adjunct professor	Human Sciences
L28	Control	M	38	Adjunct professor	Economic Sciences
L29	Control	M	49	Associate professor	Human Sciences

**Caption:** F = Female; M = Male

**Table 3:** Sociodemographic information by groups

Variable	Intervention Group (n=17)		Control group (n=12)	
	Mean	SD	Mean	SD
Age	47.5	10.4	39.8	10.8
Experience teaching (years)	17.5	10.2	8.9	5.8
Teaching hours per day	5.6	1.9	6.8	1.7
Teaching days per week	4.9	1.3	5.1	0.9
Number of students per class	25.5	8.2	27.8	10.0

**Caption:** SD = Standard deviation

### Stress levels before and after the WVHLS program

At the beginning of this study, all participants had moderate stress levels, except for male participants from the control group, who reported high-stress levels. As seen in Table 4, at the end of this study, female participants from the non-intervention group showed increased stress levels (trend towards an elevated level) compared with their pairs in the intervention group.

**Table 4:** Measurements at baseline and at the end of follow-up

		Intervention Group				Wilcoxon signed-rank test	Control Group				Wilcoxon signed-rank test
		F (n=7)		M (n=8)			F (n=6)		M (n=8)		
Self-report	Measure	Mean	SD	Mean	SD	p-value	Mean	SD	Mean	SD	p-value
Level stress	**PRE**	2.6	0.7	2.3	1.1	0.132	3.0	0.6	3.6	1.1	0.739
	**POST**	3.0	0.6	2.3	1.3		3.4	1.0	2.8	1.3	
Daily voice use	**PRE**	5.8	2.0	5.0	1.6	0.931	6.0	1.3	8.0	1.6	0.107
	**POST**	5.8	1.9	4.8	1.6		5.6	1.5	7.4	1.7	
Factor 1	**PRE**	16.1	9.2	20.0	7.5	0.190	21.7	10.5	27.4	10.2	0.563
	**POST**	13.8	7.8	15.5	9.7		21.0	13.5	23.6	3.2	
Factor 2	**PRE**	4.6	4.1	5.3	4.1	0.649	8.3	5.1	8.8	7.0	0.636
	**POST**	5.2	4.5	5.5	3.7		7.6	5.9	8.0	3.2	
Factor 3	**PRE**	9.3	2.5	7.5	3.9	0.428	10.6	1.6	9.2	2.2	0.107
	**POST**	9.8	2.2	9.3	2.1		10.3	2.4	7.2	1.8	
**Voice parameters**											
MPT (Seg)	**PRE**	13.8	3.5	13.6	4.7	0.148	15.0	4.0	18.3	3.6	0.008*
	**POST**	14.1	3.0	18.4	3.0		17.6	4.7	22.3	1.9	
Jitter (%)	**PRE**	0.32	0.08	0.45	0.20	0.408	0.33	0.14	0.34	0.12	0.875
	**POST**	0.27	0.09	0.61	0.25		0.32	0.10	0.34	0.18	
Shimmer (%)	**PRE**	6.48	4.57	4.42	1.06	0.959	6.64	5.93	4.89	1.94	1.000
	**POST**	6.19	3.54	4.89	1.88		4.80	2.80	5.83	3.77	
HNR (dB)	**PRE**	16.88	4.60	17.53	3.08	0.469	17.61	6.41	15.44	2.43	0.433
	**POST**	16.70	4.14	16.09	1.77		17.70	3.88	17.18	4.57	

* p<0.05 differences pre and post intervention

**Caption:** F = Female; M = Male; SD = Standard deviation; PRE = Before intervention; POST =After intervention

### Vocal functioning before and after the WVHLS program

Concerning voice functioning, participants reported high scores in Factors 1 - 2 and 3 of the VFI before starting the program, indicating that they already had self-perceived vocal fatigue. At the end of the follow-up, Factor 1 (“tiredness of voice and voice avoidance”) scores were lower among subjects in the intervention group compared with their pairs in the non-intervention group. However, this effect was not statistically significant (p>0.05; Wilcoxon signed-rank test).

Regarding the aerodynamic parameter, participants had a longer MPT at the end of the study compared with the baseline measures. However, all groups had shorter than normal MPT values (except for men in the non-intervention group)^(28)^. In the case of Shimmer and HNR, before and after the intervention, these parameters were not within the normal ranges^(27)^. At the end of the study, women in the intervention group had slightly longer MPT, smaller jitter, shimmer and HNR, whereas their colleagues in the non-intervention group had longer MPT, smaller jitter and shimmer, and slightly higher HNR. For participating males, at the end of the study, there was a similar tendency in both groups (intervention vs. non-intervention) with longer MPT, and increased jitter (not in the non-intervention group) and shimmer. The men in the intervention group had smaller HNR, whereas their colleagues in the non-intervention group presented the opposite tendency. However, there were no statistical differences between the intervention group and control group (p>0.05; Wilcoxon signed-rank test) (Table 4). In contrast, we found an association between the intervention WVHLS promotion program and MPT (p=0.001) and Factor 1 of VFI. (p<0.10) as can be seen in Table 5.

**Table 5:** Measurements of effect of intervention WVHLS program

	Effect of Intervention program	
Self-report VFI	Β	p-value
Factor 1	-1.131	0.073+
Factor 2	-0.055	0.600
Factor 3	0.004	0.947
**Voice parameters**		
MPT (Sec)	0.137	0.001*
Jitter (%)	-0.106	0.485
Shimmer (%)	-0.060	0.617
HNR (dB)	0.003	0.945

* p<0.05

+ p<0.10

**Caption:** F = Female; M = Male; SD = Standard deviation; B = Beta

## DISCUSSION

This research aimed to determine the effect of an online WVHLS promotion program implemented during COVID-19 pandemic on stress levels and voice functioning among college professors. Three main results were found: the WVHLS promotion program had no statistically significant effect on participants’ stress levels, after the WVHLS program, there was a tendency towards low perception of tiredness of voice and voice avoidance measured with the VFI (p=0.073), and at the end of the program, MPT was statistically significantly longer (p=0.001).

Concerning stress levels, we did not find changes at the end of the program. One explanation is related to the moment of the measurement. Since, at the end of the follow-up, professors could be exposed to high-stress levels due to the closing of the semester, this hampered the ability to identify a decrease in stress levels. A second explanation is related to the duration of Module II (Stress Management), which may have been considered as short duration and limit the effect of the proposed activities. A third explanation is related to the sample size, which was smaller than planned (and due to the COVID-19 pandemic, we were unable to recruit more participants), which may have a hamper to identify significant changes before and after and between groups. Nevertheless, although the results were not statistically significant, anecdotally, participants mentioned perceived benefits in managing stressors in their workplace when applying the strategies mentioned during the sessions.

Regarding self-perception of vocal fatigue, although all participants had lower scores for VFI Factor 1 at the end of the follow-up, there was a tendency that college professors from the intervention group had a bigger decrease (although not statistically significant) compared with their pairs in the non-intervention group. Since vocal fatigue is one of the most common voice symptoms among teachers, the tendency towards a lower Factor 1 score may suggest that teachers’ awareness of healthy occupational voice use increased. Interestingly, our results suggest more consistent changes in VFI scores compared to acoustic parameters after the implementation of the WVHLS program. One explanation is that participants had greater awareness of their vocal demand responses but their adjustments in their voice production were small, and not perceptible by voice acoustic parameters. These findings agree with Imaezue and Oyebola^(29)^ who reported that vocal hygiene training and resonant voice therapy are effective in reducing self-perceptive symptoms of vocal fatigue among teachers.

Our results on the longer MPT after the implementation of the program and notable tendency to decreased jitter and shimmer after the implementation of the program agree with the results reported by Relekar and Mukundan^(30)^, who found significant improvements in the acoustic parameters including *fo*, jitter and shimmer in secondary school teachers after the implementation of a 15-days vocal training program^(30)^. Likewise, lower jitter and shimmer indicates more stability of the voice and consequently a general increase in the participants’ voice quality. Nevertheless, there were no statistically significant differences in voice acoustic parameters between the groups of this study, which may be attributable to the small sample size. Another explanation is the 100% online design of the study, which implies high self-administration of the daily activities during the week (in the daily practice exercises) and just one synchronous meeting to review new information and provide feedback. If we keep in mind that in addition to the program, participating professors were teaching 100% online, their treatment adherence may have been low (low daily practice), which may have influenced the low/minor changes. We did not evaluate adherence in our study, but future studies are needed to evaluate this hypothesis.

Some limitations should be considered on the interpretation of the results and improved in future studies. First, we designed the intervention program under the virtual modality, which could be a limitation for monitoring the performance of the program exercises. Second, weekly sessions with autonomous daily practice exercises were designed but no meetings were performed during the week, which may be needed considering that no statistically significant differences were found. Third, when the final measurements of this study were collected, participants were at the end of the semester, so they could have been very stressed by the increase in the workload.

## CONCLUSION

In conclusion, the results of this study, although not statistically significant, suggest a positive effect of a WVHLS program on reducing vocal fatigue perception measured by means of the Vocal Fatigue Index, and improving coordination and control of breathing speech measured MPT. Although, the small sample size as well as the 100% online design may have influenced the lack of statistically significant associations, these changes at the end of the follow-up may indicate that holistic programs that include voice care recommendations, breathing exercises, vocal warm-up and cold-down exercises, laryngeal relaxation vocal exercises and stress management may be beneficial for reducing work-related stress and voice symptoms among college professors. Follow-up studies with bigger sample sizes and hybrid designs (50% online and 50% in-person) are advised to determine the effect of Workplace Health programs among teachers.
